# Cell Wall Antibiotics Provoke Accumulation of Anchored mCherry in the Cross Wall of *Staphylococcus aureus*

**DOI:** 10.1371/journal.pone.0030076

**Published:** 2012-01-10

**Authors:** Wenqi Yu, Friedrich Götz

**Affiliations:** Microbial Genetics, University of Tübingen, Tübingen, Germany; Monash University, Australia

## Abstract

A fluorescence microscopy method to directly follow the localization of defined proteins in *Staphylococcus* was hampered by the unstable fluorescence of fluorescent proteins. Here, we constructed plasmid (pCX) encoded red fluorescence (RF) mCherry (mCh) hybrids, namely mCh-cyto (no signal peptide and no sorting sequence), mCh-sec (with signal peptide), and mCh-cw (with signal peptide and cell wall sorting sequence). The *S. aureus* clones targeted mCh-fusion proteins into the cytosol, the supernatant and the cell envelope respectively; in all cases mCherry exhibited bright fluorescence. In staphylococci two types of signal peptides (SP) can be distinguished: the +YSIRK motif SP_lip_ and the −YSIRK motif SP_sasF_. mCh-hybrids supplied with the +YSIRK motif SP_lip_ were always expressed higher than those with −YSIRK motif SP_sasF_. To study the location of the anchoring process and also the influence of SP type, mCh-cw was supplied on the one hand with +YSIRK motif (mCh-cw1) and the other hand with -YSIRK motif (mCh-cw2). MCh-cw1 preferentially localized at the cross wall, while mCh-cw2 preferentially localized at the peripheral wall. Interestingly, when treated with sub-lethal concentrations of penicillin or moenomycin, both mCh-cw1 and mCh-cw2 were concentrated at the cross wall. The shift from the peripheral wall to the cross wall required Sortase A (SrtA), as in the *srtA* mutant this effect was blunted. The effect is most likely due to antibiotic mediated increase of free anchoring sites (Lipid II) at the cross wall, the substrate of SrtA, leading to a preferential incorporation of anchored proteins at the cross wall.

## Introduction

Surface anchored proteins of *Staphylococcus aureus* represent a group of proteins that are exposed on the bacterial cell envelope and covalently anchored to the staphylococcal cell wall peptidoglycan [1]. Many of the surface proteins belong to the MSCRAMM family (microbial surface components recognizing adhesive matrix molecules), which play key roles in colonization and adhesion of *S. aureus* [2].

The process of anchoring surface proteins to the staphylococcal cell wall, termed the ‘sorting pathway’, includes three steps [3]: translocation, sorting and incorporation into mature peptidoglycan. Anchored proteins are distinguished by a C-terminal cell wall sorting signal (CWS). The N-terminal signal peptide directs the polypeptide into the Sec secretory translocon. Sortase A (SrtA) [4], a membrane-bound transpeptidase, performs the sorting reaction by cleaving the amide bond between threonine and glycine within the LPXTG motif, which results in the acyl intermediate. The peptidoglycan precursor, Lipid II, serves as the substrate for the sorting reaction, which is the tethering of the C-terminal threonine of the surface protein to lipid II by an amide bond. Lipid II tethered with the surface proteins is finally incorporated into mature peptidoglycan [5].

Previously, we have described that the N-terminal signal peptides of staphylococcal lipases harbor a conserved motif - Ser, Ile, Arg and Lys - designated as the SIRK-motif [6]. This motif (termed as YSIRK/GS) is later found conserved in many, but not all surface proteins. SP with the YSIRK/GS motif promotes the secretion of surface proteins [7]. In *Streptococcus pyogenes* [8] and in *S. aureus* [9], the SP (+YSIRK-motif) has a function in directing surface proteins to different surface localizations. In *S. aureus*, SP (+YSIRK) directs the secretion and anchoring of surface proteins at septum (cross wall), while the SP (−YSIRK) leads the secretion and anchoring of surface proteins more to the cell pole [9]. It has also been shown that three transmembrane proteins, namely Spd (surface protein display) proteins, are involved in the surface display of protein A, one of the predominant surface proteins carrying SP (+YSIRK) [10]. The expression level and surface display of protein A are largely reduced in each *spd* mutant. Moreover, *spd* mutants affect the expression of surface proteins with SP (+YSIRK). Interestingly, the *spd* mutants exhibit an increased abundance of visible cross walls and thickened cross walls. Yet, how cross wall formation affects the surface display of surface proteins remains unclear.

Conventionally, immunofluorescence microscopy has been applied to surface proteins localization studies, as the cell surface immobilized proteins have relatively easy and stable access to antibodies [11], [12]. However, immunofluorescence microscopy has a certain intrinsic limitation that especially impedes the subcellular and high throughput studies. For example, antibodies cannot penetrate into the septum without cell wall permeabilization; yet cell wall permeabilization using cell wall hydrolase or detergents often leads to the release of surface proteins with the risk of artifacts. Further, a large numbers of specific antibodies are needed in order to study various surface proteins' localization, which is laborious and time consuming. Particularly in *S. aureus* immunofluorescence is extremely hindered by protein A, the IgG binding protein.

In this study, we developed a direct visualization method for monitoring the surface proteins anchoring process. The red fluorescent protein mCherry was fused with different signal sequences and targeted as cytoplasmic, secreted, and cell wall anchored. Cell wall anchored mCherry (mCh-cw) enabled us to visualize the cross and peripheral wall localization pattern rather than using immunofluorescence microscopy. Intriguingly, independent of different signal peptides, treatment with sub-lethal concentrations of cell wall biosynthesis antibiotics led to strong accumulation of mCh-cw at the cross wall which correlated with the increased Van-FL binding at the cross wall. Our results show that mCherry is a useful tool to localize and follow the anchoring or secretion processes in staphylococci.

## Results

### Defined mCh-fusion proteins are targeted in an active form (maintaining RF) to distinct subcellular compartments

**Figure 1:**
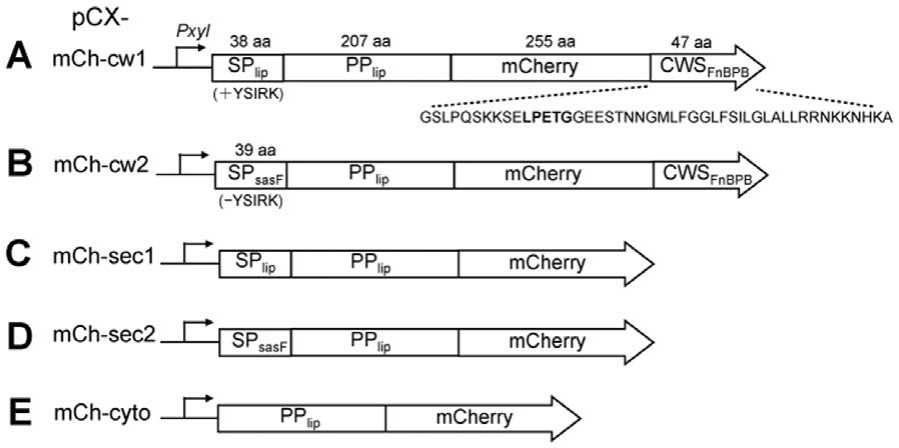
Schematic representation of mCh-hybrids. SP, signal peptide; PP, propeptide; CWS, cell wall sorting signal; mCh: mCherry; lip, lipase. The amino acid sequence of CWS was indicated. The parent plasmid was pCX30 and all mCh-fusion constructs were under control of the xylose-inducible promoter, P*xyl*.

Previously, we have anchored staphylococcal lipase to staphylococcal cell wall in an active form [13]. Anchored lipase could be extracted from the cell wall, together with covalently tethered peptidoglycan [14]. Based on these results, we asked if mCherry could be immobilized to staphylococcal peptidoglycan while maintaining stable fluorescence. The mature lipase was replaced by mCherry in pCX30Δ82, generating pCXmCh-cw1 (**Fig. 1A**). The protein domain order in this construct was, the N-terminal signal peptide (SP_lip_) and propeptide (PP_lip_) of lipase, mCherry, and the C-terminal cell wall sorting sequence (CWS) of FnBPB (fibronectin binding protein B). CWS consisted of the LPXTG motif, followed by a hydrophobic domain and a positively charged tail [13]. To differentiate the effect of SP (+/−YSIRK), the signal peptide of surface protein SasF (SP_sasF_), a non-YSIRK SP was used to substitute SP_lip_, resulting in pCXmCh-cw2 **(** **Fig. 1B** **)**. Moreover, hybrids mCh-sec1&2 lacking C-terminal CWS, as well as hybrid mCh-cyto lacking both SP and CWS, were constructed **(** **Fig. 1C, 1D, 1E** **)**. All the fusions were carried out under the xylose inducible and glucose repressible *Pxyl* promoter of the pCX30 vector backbone [15]. Importantly, it was necessary to keep the PP_lip_ in the fusion with mCherry in all the constructs, since PP_lip_ significantly promotes the fusion partners' secretion, stability and activity [16], [17]. Expressing mCherry without PP_lip_ showed drastically reduced fluorescence (data not shown).

**Figure 2:**
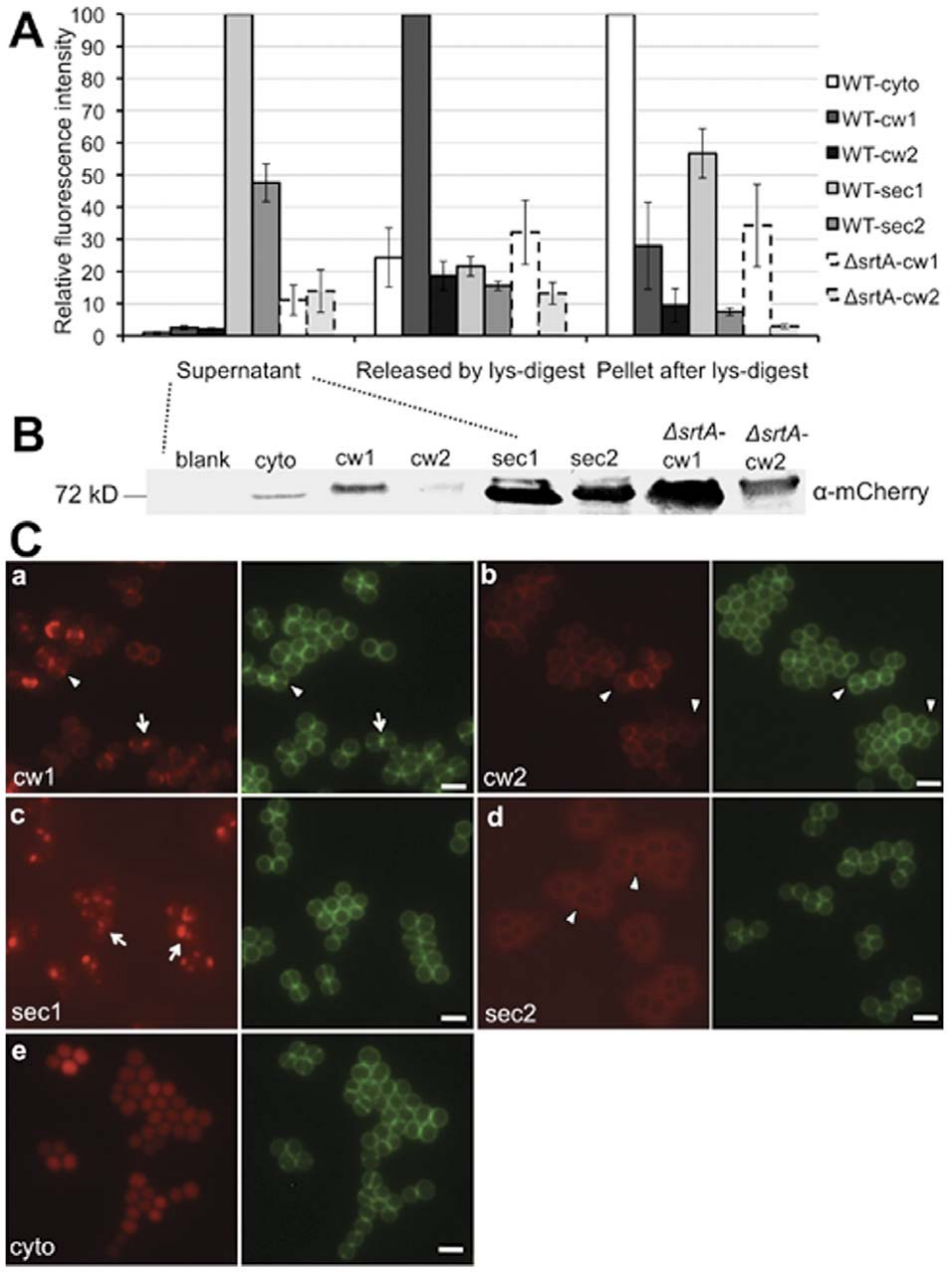
Monitoring mCh-hybrids. **A**. Fluorescence intensity comparison of mCh-hybrids from different cell fractions. WT-cyto, SA113 (pCXmCh-cyto); WT-cw1 or 2, SA113 (pCXmCh-cw1) or (pCXmCh-cw2); WT-sec1 or 2, SA113 (pCXmCh-sec1) or (pCXmCh-sec2); Δ*srtA*-cw1 or 2, SA113 Δ*srtA* (pCXmCh-cw1) or (pCXmCh-cw2); lys, lysostaphin. **B.** Western blotting of mCh-hybrid proteins in the culture supernatant of protein A deficient mutant SA113 Δ*spa*. Blank, SA113 Δ*spa* without plasmid; cyto, SA113 Δ*spa* (pCXmCh-cyto); cw1 or 2, SA113 Δ*spa* (pCXmCh-cw1) or (pCXmCh-cw2); sec1 or 2, SA113 Δ*spa* (pCXmCh-sec1) or (pCXmCh-sec2); Δ*srtA*-cw1 or 2, SA113 Δ*spa*Δ*srtA* (pCXmCh-cw1) or (pCXmCh-cw2). **C.** Subcellular localization of mCh-hybrid proteins in SA113. **a.** pCXmCh-cw1; **b.** pCXmCh-cw2; **c.** pCXmCh-sec1; **d.** pCXmCh-sec2; **e.** pCXmCh-cyto. Arrowheads in **a** and **b**, fluorescence localized at the cross wall in **a**, but absent from the cross wall in **b**; arrows in **a** and **c,** RF foci close to the initial sites of the cross walls; arrowheads in **d,** halo-like RF distribution absent from the cross wall. Images **a, c,** and **e** were taken after one hour of xylose induction; images **b** and **d** were taken after two hours of induction. Green: Van-FL staining (cell wall); scale bar, 2 µm.

To test if mCh-hybrids were functional, the plasmids were transformed into *S. aureus* SA113 (WT) and its SrtA mutant (Δ*srtA*). After xylose induction, different cell fractions were collected for mCherry expression (indicated by RF measurement). As shown in **Fig. 2A**, the supernatant of WT-sec1 exhibited the highest RF signals, which were set as 100%. WT-sec2 showed the second highest RF intensity of about 50%. WT-cw1, WT-cw2 and WT-cyto had little RF in the supernatant**,** while the Δ*srtA*-cw1 or Δ*srtA*-cw2 showed 10–15% RF intensity **(** **Fig. 2A** **)**. All constructs (anchored or secreted mCh-hybrids) with SP_lip_ (+YSIRK motif) exhibited significantly higher fluorescence intensity than those with SP_sasF_ (-YSIRK motif). The same results were obtained in SA113 Δ*spa* (data not shown), where the protein levels could be accessed by Western blotting without the interference of protein A. The protein level of different constructs **(** **Fig. 2B** **)** correlated with their fluorescence profiles, except for Δ*srtA*-cw1 and Δ*srtA*-cw2 where mCh-cw was released into the supernatant with the unprocessed C-terminal CWS. Possibly, the unprocessed CWS interfered with the correct folding of mCherry; therefore, the fluorescence emission was reduced to some extent. Once covalently anchored to peptidoglycan, surface proteins are immobilized and can only be released by peptidoglycan hydrolyses [3], [13]. Lysostaphin, the glycyl-glycine endopeptidase, cleaves specifically the pentaglycine cross bridges in staphylococcal peptidoglycan, and thereby releases the surface proteins that are linked to pentaglycine bridges. WT-cw1 released the highest amount of RF by lysostaphin treatment, indicating that mCherry was largely peptidoglycan-immobilized. In WT-cw2 five-fold less RF was released **(** **Fig. 2A** **)**. In the pellet fraction after lysostaphin treatment, WT-cyto displayed the highest fluorescence, indicating that without SPs, mCh-fusion proteins were not secreted but remained in the cytosol **(** **Fig. 2A** **)**. SA113 WT (pCX30Δ82) showed no fluorescence in all cell fractions, like the negative controls, which were SA113 without plasmid or the BO medium (data not shown). To test if mCh-hybrids were functional in different staphylococcal species, all constructs were transformed into *S. carnosus* TM300 and its *srtA* deletion mutant; we obtained similar results as with *S. aureus* strains (data not shown).

### mCh-hybrids provide useful tools to visualize the effect of SP (+/− YSIRK-motif)

In earlier studies it was suggested that SP (+YSIRK) directs the secretion and anchoring of surface proteins at the division septum, whereas the surface proteins with SP (−YSIRK) are secreted and incorporated at the cell pole [8], [9]. To test if the spatial difference of SP (+/−YSIRK) can be visualized by mCh-hybrids, we compared the mCh-fusions with SP_lip_ (+YSIRK) and SP_sasF_ (−YSIRK). Indeed, the localization patterns of the SA113 (pCXmCh-cw1) and SA113 (pCXmCh-cw2) clones differed from each other. The mCh-cw1 clone exhibited patchy circumferential RF and especially bright RF at the cross wall (**Fig. 2C** **a, arrowheads**); often, two foci adjacent to the new cross wall were observed **(** **Fig. 2C** **a, arrows).** In contrast, in the mCh-cw2 clone RF distributed homogeneously at the peripheral cell wall; little RF was seen in the cross wall, even after two daughter cells split **(** **Fig. 2C** **b, arrowheads).** Quantification of colocalization analysis of Van-FL (green fluorescence of cell wall staining) and mCh-cw (RF) revealed that mCh-cw1 colocalized with nearly 50% of the total cross walls, while mCh-cw2 colocalized with only 6% of total visible cross walls **(Fig. S1B)**.

The effect of SPs (+/−YSIRK) can also be visualized by the secretion patterns of SA113 (pCXmCh-sec1) and SA113 (pCXmCh-sec2). In mCh-sec2, most of RF was outside and surrounding the cells as a diffuse halo while absent at the cross walls **(** **Fig. 2C** **d, arrowheads)**, which indicated a peripheral secretion pattern. In contrast, mCh-sec1 exhibited spot-like foci particularly at or near the (future) division septum **(** **Fig. 2C** **c, arrows)**. The different localization pattern between SA113 (pCXmCh-sec1) and SA113 (pCXmCh-sec2) was in agreement with earlier observations that SPs (+/−YSIRK) probably direct the secretion of surface proteins to different sites [8], [9]. In the cytoplasmic expressed mCh-hybrids of SA113 (pCXmCh-cyto), RF was uniformly distributed within the cells **(** **Fig. 2C** **e)**.

### Penicillin and moenomycin direct mCh-cw to the cross wall, irrespective of SP type

**Figure 3:**
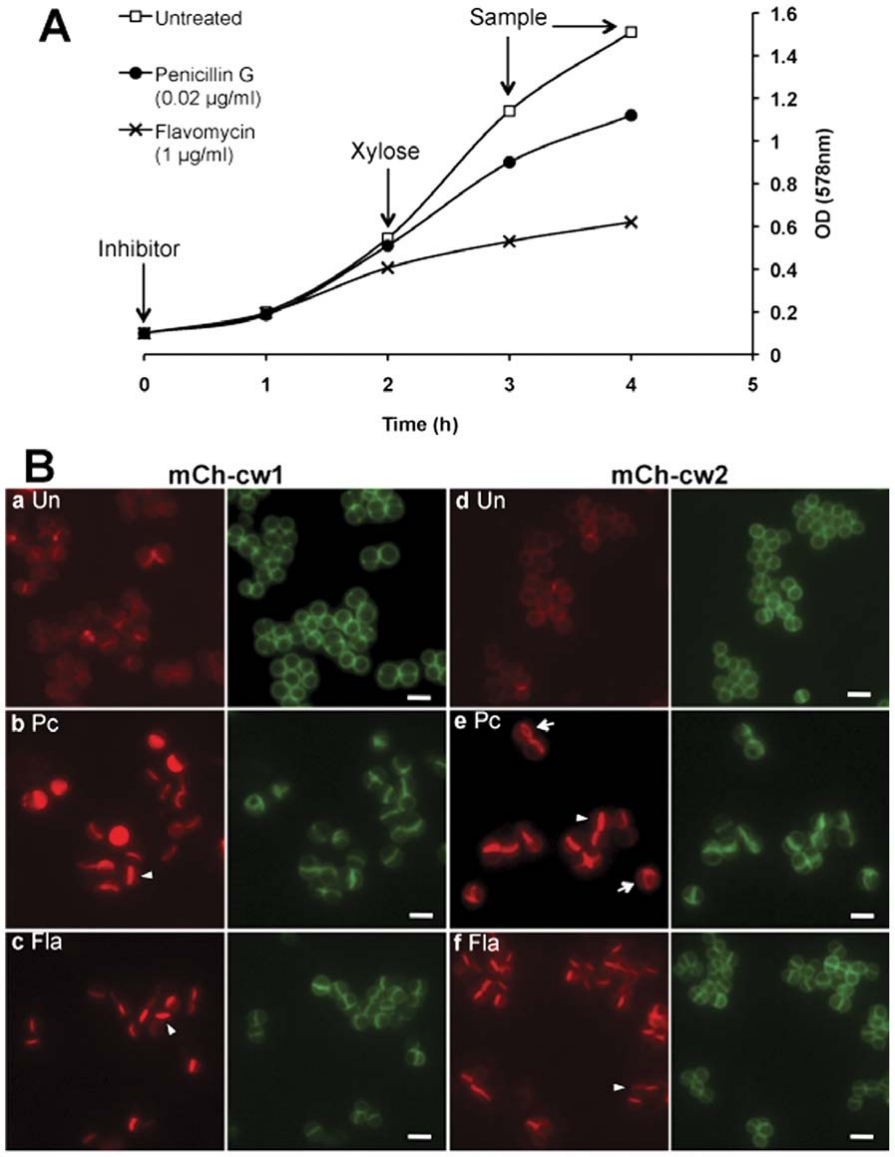
Penicillin and moenomycin direct mCh-cw to the cross wall, irrespective of SP type. **A.** Schematic representation of antibiotics treatment assay. Untreated (**□**); treated with penicillin (0.02 µg/ml) (•); treated with moenomycin (flavomycin) (1 µg/ml) (×). **B.** Influence of penicillin (Pc) and moenomycin (Fla) on the subcellular localization of mCh-cw hybrid proteins. Arrowheads indicated the cross wall accumulation of mCh-cw; arrows indicated the ring-like distribution; scale bar, 2 µm.

Several cell wall biosynthesis antibiotics interfere with the protein anchoring reaction [5], [18]. It has been shown that for example penicillin G, vancomycin, moenomycin, bacitracin and tunicamycin inhibit the tethering of surface proteins with lipid II. Considering that the surface proteins anchoring process is closely related to both protein secretion and cell wall biosynthesis, we examined whether these cell wall antibiotics effect the localization of secretion or anchoring. Gallidermin [19], a lantibiotic that specifically binds to lipid II [20], and D-cycloserine, which prevents D-Ala-D-Ala terminus synthesis of the muropeptides [21], were also tested. As shown in **Fig. 3A**, overnight cultures of SA113 (pCXmCh-cw) were diluted into fresh BO medium. Antibiotics were added at 0.1 OD_578_, followed by two hours of incubation before xylose induction. Samples for microscopy examination were collected after one and two hours of xylose induction. The sub-lethal concentrations of various antibiotics were determined experimentally when the bacterial growth was slightly retarded but not completely inhibited, allowing protein synthesis to proceed.

Among all of the antibiotics tested, penicillin G (Pc) and moenomycin (synonym: flavomycin, Fla) triggered a strikingly altered localization pattern compared to the untreated cells. In **Fig. 3B** the results of two hours' xylose induction were shown (the results of one hour xylose induction looked essentially the same, only fluorescence intensity was less). The most striking difference between untreated (**Fig. 3B** **a,d**) and penicillin G or moenomycin treated clones (**Fig. 3B** **b,c,e,f, arrowheads**) was that mCh-cw became almost exclusively localized at the cross wall. The antibiotics provoked an accumulation of anchored mCherry in the cross wall of *S. aureus*.

### Penicillin and moenomycin also cause Van-FL accumulation at the cross wall

In the presence of penicillin or moenomycin, we found that not only mCh-cw but also Van-FL that recognizes free -D-Ala-D-Ala of lipid II or uncrosslinked murein in the cell wall was accumulated at the cross wall while simultaneously disappearing from the side wall. We tried to quantify the percentage of Van-FL stained cross wall and the rate of cross wall localized mCh-cw (RF) in antibiotic treated and untreated cells (**Fig. S1**). The percentage of visible cross walls was the ratio of visible cross wall numbers (when Van-FL staining appeared as a line at the septum before daughter cells split) in a cell population versus the total cell numbers of the same cell population. Percentage of cross wall localized RF was the ratio of numbers of line-like cross wall localized RF versus line-like cross walls (visible by Van-FL staining) in the same cell population. Both penicillin and moenomycin treatment led to a significantly higher percentage of visible cross wall formation and an increased percentage of RF localizing at the cross wall in SA113 carrying either pCXmCh-cw1 or pCXmCh-cw2 (**Fig. S1A, B**). The effect was more pronounced in the mCh-cw2 clone. In the untreated cells, mCh-cw2 colocalized with only 6% of the cross walls, while in penicillin or moenomycin treated cells, the percentage rose to 76% and 95% respectively, implying that mCh-cw2 colocalized with almost every visible cross wall in moenomycin treated cells.

**Figure 4:**
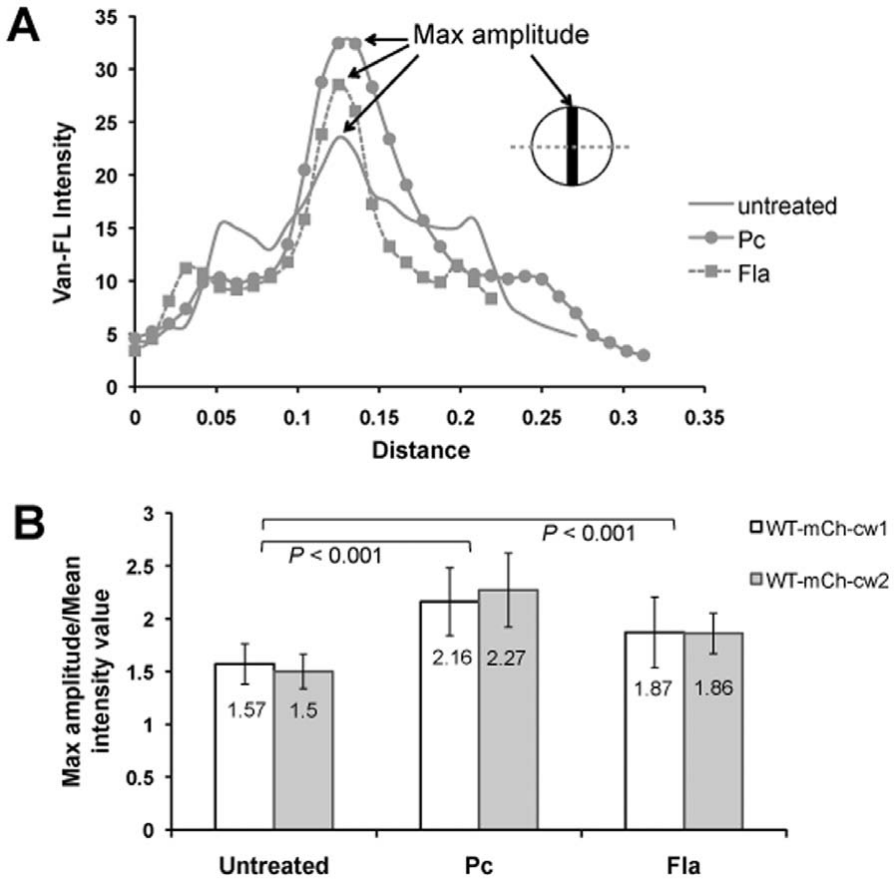
Penicillin and moenomycin treatment led to enrichment of Van-FL at the cross wall. **A.** Fluorescence intensity profile of Van-FL staining from a line perpendicular to the cross wall and across the middle point of the cross wall. Simple line, untreated cell; dotted line with filled squares, moenomycin (Fla) treated cell; line with filled circles, penicillin (Pc) treated cell. Max amplitude represented the cross wall intensity. Note that the figure was remade using ImageJ software from the microscopy images; the intensity and distance values were not the same as the original data from Leica AF software; but represented the same profile distribution. **B.** Comparative Van-FL intensity at the cross wall among untreated, penicillin (Pc) treated, and moenomycin (Fla) treated cells. The cross wall Van-FL intensity values were calculated by the ratio of max amplitude against mean fluorescence intensity (generated by Leica AF software) from the same cell. The average ratio of 150 cells from three independent experiments of each group was shown in the bars. White bar, SA113 (pCXmCh-cw1); gray bar, SA113 (pCXmCh-cw2).

The relative fluorescence intensity of Van-FL at the cross wall was also quantified. The fluorescence profile of a line that is perpendicular to the cross wall and across its middle point was compared between untreated and antibiotics treated cells (**Fig. 4A**). Only cells with a ‘cross wall line’ (a closed septum before cell split) were measured. The max amplitude (the major peak) indicated the fluorescence intensity at the cross wall. The two small peaks indicated the peripheral (side) wall fluorescence intensity. Generally, penicillin- or moenomycin-treated cells displayed higher fluorescence (RF and VanFL) at the cross wall and lower fluorescence at the peripheral wall when compared to untreated cells (**Fig. 4A**). To quantify the significance and avoid the error of staining or imaging difference, the value of max amplitude was divided by the mean Van-FL fluorescence intensity value of the same cell. **Fig. 4B** showed the average ratio (cross wall intensity/mean intensity) of 150 cells from three independent experiments in each group. The data showed that both penicillin and moenomycin significantly intensified Van-FL staining at the cross wall compared to the untreated cells. Of all cell wall antibiotics tested, penicillin and moenomycin induced the most obvious phenotype. Bacitracin and gallidermin triggered the accumulation of mCh-cw at the cross wall to a certain extent, whereas vancomycin and D-cycloserine had little influence. Under all situations, an increased Van-FL staining at the cross wall correlated with an increased mCh-cw colocalization.

In penicillin or moenomycin treated cells, Van-FL staining at the cross wall was significantly higher than that in the untreated cells, indicating that free D-Ala-D-Ala residues were enriched, which resulted from a decrease in murein cross-linking and an increase of lipid II molecules. In both scenarios, uncross-linked pentaglycines (SrtA substrates), the anchoring sites for mCh-cw, should also be increased. Thus, we assume that the increased availability of anchoring sites favors the anchoring of surface proteins, thus causing the observed incorporation and accumulation at the cross wall. This assumption was confirmed by the finding that antibiotic driven accumulation of mCh-cw at the cross wall required SrtA.

### Antibiotic induced accumulation of mCh-cw at the cross wall requires SrtA

As shown above, penicillin and moenomycin impelled the accumulation of mCh-cw at the cross wall, irrespective of SP type. The question is: does the accumulation require SrtA mediated anchoring? To verify this question, we examined the influence of penicillin and moenomycin on Δ*srtA* (pCXmCh-cw) as well as SA113 (pCXmCh-sec).

**Figure 5:**
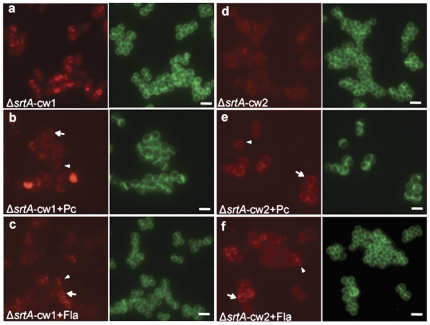
Localization patterns of Δ*srtA* (pCXmCh-cw1&2) in the presence of penicillin or moenomycin. Arrows, mCh-cw dispersed over the entire cell; arrowheads, the cross wall localized mCh-cw. Scale bar, 2 µm.

In Δ*srtA* (pCXmCh-cw), mCh-cw cannot be anchored to the cell wall due to the absence of SrtA; therefore, mCh-cw was partially released into the supernatant and partially retained in the membrane via the C-terminal CWS domain. In the presence of penicillin or moenomycin, mCh-cw was largely dispersed over the entire cell wall (both cross wall and side wall), irrespective of the SP-types **(** **Fig. 5** **)**. There was no RF accumulation at the cross wall as was seen for the SA113 WT (**Fig. 4B**), indicating that SrtA was necessary for the accumulation of mCh-cw at the cross wall.

## Discussion

So far, immunofluorescence microscopy and immunoelectron microscopy have been used for surface proteins localization studies in the last decades. To our knowledge, there is no direct visualization method to be applied in this field yet. In this study, we aimed to develop a direct method for monitoring surface proteins' subcellular distribution. The recently developed fluorescent protein mCherry, the monomeric derivative of *Discosoma* sp. fluorescent protein ‘DsRed’ [22], provided us with an ideal tool. mCherry was found fully fluorescent after secretion through the Sec secretory pathway and was fluorescent in the membrane as well [23], [24]. Here we show that mCherry can be secreted and anchored to staphylococcal cell wall while maintaining stable fluorescence.

Our trial with GFPmut3 [25] failed, because GFPmut3 lost fluorescence when it was translocated via the Sec secretory pathway **(Fig. S3A)**, similar to the observation with GFPuv in *Escherichia coli* [26]. It has been reported recently that a new GFP variant, the super-folder GFP (sfGFP) [27], can be translocated through the Sec secretory pathway in *E. coli* while maintaining fluorescence [28]. However, in *S. aureus*, the fluorescence of secreted sfGFP-fusions was still fairly low, although the sfGFP-fusions were secreted in a higher amount than the GFPmut3-fusions **(Fig. S3)**. In comparison, the secreted mCh-fusions showed 7–13 fold higher fluorescence intensity than GFP-fusions while the difference in the protein amount was not that remarkable **(Fig. S3)**. Western blotting results revealed that different from the secreted mCh-fusions, the secreted GFP-fusions (ppGFP-sec1, ppGFP-sec2, ppsfGFP-sec1, and ppsfGFP-sec2) migrated slightly higher than ppGFP or ppsfGFP **(Fig. S3B, arrows)**, which indicated that the majority of the secreted GFP-fusions were still tethered with signal peptides. It appeared that the secreted GFP-fusions could not be processed and fold correctly to be fluorescent after Sec-dependent secretion. The mCh-hybrids constructed in this study enabled us to observe and follow the subcellular (especially the cross wall) localization of anchored proteins. Meanwhile, we were also fully aware of the limitation of the system, as it was based on plasmid-encoded genes, by which the proteins were higher expressed. Yet, prolonged protein expression only enhanced the fluorescent signals; it did not alter the distribution patterns within the time period tested, one and two hours after induction. Therefore, we can make at least statements as to the tendency of protein localization.

Apart from the influence of SPs (+/−YSIRK) on the localization of secretion, we also found that in the presence of the YSIRK-motif the RF intensity of mCh-fusion proteins was significantly increased. As shown in **Fig. 2C** **c,** mCh-sec1 exhibited spot-like bright foci at or near the division septum, which very likely resulted from the highly expressed proteins that exceeded the capability of protein transport. Indeed, mCh-sec1 showed higher RF than mCh-sec2 in both the supernatant and the cell pellet **(** **Fig. 2A** **)**, implying that mCh-sec1 was expressed in a higher amount than mCh-sec2. The tendency that proteins fused with SP_lip_ (+YSIRK) were always higher expressed was observed in all mCh-constructs as well as in all GFP-fusions **(Fig. S3B**). The difference in protein expression was most likely due to different SPs, as the plasmid, promoter, and RBS were identical in all constructs. Whether transcriptional or post-transcriptional regulation was responsible for the positive effect of the SP (+YSIRK) needs to be verified. In principle, we could confirm earlier results that *S. aureus* distinguishes between SPs to either direct (+YSIRK)-proteins to the cross wall (cell division site) or (-YSIRK)-proteins to the side wall [9]. How the targeting is accomplished is unknown, but one cannot rule out that the different targeting is due to the different expression levels of +/−YSIRK-motif proteins. It is worthwhile to investigate the influence of expression rate on targeting.

One of the most interesting findings of our study was the effect of sub-lethal concentrations of penicillin or moenomycin. These two antibiotics provoke concentration of cell wall-anchored mCh-cw1&2 at the cross wall, irrespective of their SP-type (**Fig. 3B**). The antibiotics also had an effect on secreted mCh-sec1&2; here it looked as if the release of mCh-sec was retarded, leading to an accumulation at or near cross wall sites of the cell envelope (**Fig. S2**). We also addressed the question of which role SrtA might play in targeting. In Δ*srtA,* proteins remain at least transiently in the membrane via their C-terminal CWS domain. In the absence of antibiotics a similar distribution of mCh-cw was observed in Δ*srtA,* as in WT. In Δ*srtA-*mCh-cw1, mCh was more accumulated in the cross wall and in Δ*srtA-*mCh-cw2, mCh was more abundant in the side wall (**Fig. 5a,d**). The effect of penicillin and moenomycin in the Δ*srtA* mutant was, however, not as pronounced as in the WT.

In the presence of penicillin or moenomycin, not only mCh-cw but also Van-FL was concentrated in the cross wall, indicating that there is an increased content of free D-Ala-D-Ala residues (e.g., uncross-linked pentaglycine bridges or lipid II molecules), which represent the substrates for the SrtA transpeptidation reaction. Such an accumulation of uncross-linked peptidoglycan precursors can be postulated since penicillin and moenomycin are known to bind to the active site of PBPs, thus blocking the transpeptidation and transglycosylation, respectively [29], [30]. It was surprising that vancomycin had little effect on mCh-cw distribution, as theoretically vancomycin inhibits both transpeptidation and transglycosylation. The previously described inhibiting effect of vancomycin is most likely due to the 10-times higher concentration used in their studies causing a complete inhibition of transpeptidation or transglycosylation [5], [18].

This paper is more than the introduction of a new experimental approach. We used this new tool to directly follow the targeting and anchoring of various mCh-hybrid constructs. We found that the SPs with or without YSIRK motif targeted proteins to different subcellular localizations. However, in the presence of sub-lethal concentrations of penicillin and moenomycin the influence of SP in targeting was abrogated as all anchored mCh-cw was concentrated at the cross wall. We assume that the antibiotics cause accumulation of SrtA substrates at the cross wall, which attract SrtA to incorporate the mCh-cw almost exclusively at the cross wall, irrespective of SP type. With this study we contribute to better understanding the influence of different signal peptide types in targeting anchored and secreted proteins and the role of cell wall antibiotics.

## Materials and Methods

### Bacterial stains and growth conditions

The bacteria strains used were *S. aureus* SA113, SA113 Δ*srtA* [31], *S. carnosus* TM300, and *S. carnosus* TM300 Δ*srtA* [32]. To perform Western blotting analysis und avoid the interference of protein A in SA113 Δ*srtA*, SA113 Δ*spa*Δ*srtA* was generated by transducing Δ*srtA*::erm to a marker-less SA113 Δ*spa* strain (this study). Generally, pre-cultures of staphylococci were cultivated at 37°C in Basic Medium (BM) composed of 1% peptone, 0.5% yeast extract, 0.5% NaCl, 0.1% glucose and 0.1% K_2_HPO_4_. Overnight pre-cultures were diluted to OD_578_ = 0.1 in fresh BO medium (BM without glucose); 0.5% xylose was added at OD_578_ = 0.5 to induce genes' expression, if not stated specifically. When necessary, cultures were supplemented with chloramphenicol 10 µg/ml (Sigma), erythromycin 5 µg/ml (Sigma).

### Construction of plasmids

Standard techniques were used for DNA manipulation and polymerase chain reaction (PCR) [33]. Electroporation of staphylococci was performed as described previously [34]. Plasmids isolation and DNA fragments purification were done using commercial kits from Qiagen. Enzymes used to manipulate DNA were from New England BioLabs or Fermentas. Oligonucleotides were synthesized from biomers.net GmbH (Ulm, Germany). DNA sequencing was performed by GATC Biotech AG (Konstanz, Germany).

The backbone for plasmid construction was pCX30 and its derivatives pCX30Δ82 [13]. pCXmCh-cw1 was constructed by the replacement of the mature *lipase* gene fragment with *mCherry* in pCX30Δ82. The *mCherry* gene without stop codon was amplified from plasmid pJCL61 (a gift from P. L. Graumann) by using primers mch1 (ATACCGCCTAGGATGGTGAGCAAGGGCGAGGAGGATA) and mch2 (TTATGCAAGCTTCCCTTGTACAGCTCGTCCATGCCGCCGGT). The PCR product was digested with AvrII-HindIII and cloned into pCX30Δ82, resulting in an in-frame fusion of mCherry with the N-terminal lipase signal peptide (SP_lip_) and propeptide (PP_lip_), together with the C-terminal cell wall sorting sequence (CWS). To construct pCXmCh-cw2, the signal peptide sequence of *sasF* (*SP_sasF_*) was amplified from the chromosomal DNA of SA113 by using primers mch3 (CGCGGATCCGAGGAGGTTTAATTAATGTTGATGGCTAAATATCGAGGGAAACCGTTT) and mch4 (CTCGCATGCAGCTTGGGCATCGTACGGCAAGATATTC). Primers mch5 (CTCGCATGCAATGATTCGACAACACAAACAACGA) and mch2 were used to amplify *pp-mCherry* fragment from pCXmCh-cw1. The PCR products of *SP_sasF_* and *pp-mCherry* were digested with SphI and ligated together. The ligation mixture was used as the template for another round of PCR using primers mch3 and mch2 to produce the *SP_sasF_-pp-mCherry* fusion. *SP_sasF_-pp-mCherry* fusion was restricted by BamHI-HindIII and cloned into the same digested pCX30Δ82. To construct pCXmCh-cyto, the *pp-mCherry* fragment containing the Shine-Dalgarno sequence and the stop codon was amplified from pCXmCh-cw1 by primers mch6 (TATGCGGATCCTATCTAGGAGGTATTAATTATGAATGATTCGACAACACAAACAACGACA) and mch7 (TTATGCTCTAGACTACTTGTACAGCTCGTCCATGCCGCCGGT), digested with BamHI-XbaI and ligated with BamHI-XbaI restricted pCX30Δ82. To construct pCXmCh-sec1, the DNA fragment of *mCherry* amplified by primers mch1 and mch7 was restricted with AvrII-XbaI and cloned into pCX30Δ82. pCXmCh-sec2 was constructed by digesting *SP_sasF_-pp-mCherry* PCR product from primers mch3 and mch7 with BamHI-XbaI, and subsequent ligation with similarly digested pCX30Δ82.

### Enzymatic release, fluorescence measurement and Western blotting

Cultures of *S. aureus* SA113 harboring pCX30Δ82 and mCh-hybrid plasmids were un-induced (as control) and induced with xylose followed by two hours of continued growth. Cells were harvested by centrifugation at 13,000×*g* for 15min. Supernatant was filtered before fluorescence measurement. Cell pellets were washed three times with Tris buffer (50 mM Tris, 150 mM NaCl, pH 7.5). Afterwards, cells were resuspended in Tris buffer supplemented with 0.5 M sucrose and normalized to the same OD_578_ = 1. 200 µl of the cell suspensions were treated with 25 µg/ml lysostaphin (Genmedics, Reutlingen, Germany) at 37°C for 10 min followed by immediate centrifugation at 13,000×*g* for 15min. The supernatant and the cell pellet after digestion were collected separately for fluorescence measurement. mCherry's RF signals were measured at 580nm excitation and 630nm emission wavelength by Tecan infinite 200 Microplate Reader (Tecan Group Ltd., Männedorf, Switzerland). SA113 without plasmids or the BO medium served as negative controls. The fluorescence intensity of supernatant was divided by the OD of the harvesting time. To perform the Western blotting analysis, mCh-hybrids were transformed into SA113 Δ*spa* and SA113 Δ*spa*Δ*srtA.* After the same induction procedure as for SA113 WT, the filtered culture supernatant was collected and normalized according to the OD of the harvesting time. Proteins were precipitated with 10% trichloroacetic acid (TCA), washed in acetone and dried in SpeedVac for 1 min. The pellet was resuspended in 1× loading buffer for SDS-PAGE and Western blotting. Hybrid proteins were detected by a rabbit polyclonal anti-mCherry antibody (Antibodies-online GmbH, Aachen, Germany).

### Antibiotics treatment and growth curve monitoring

To optimize the concentration of each antibiotic used in this study, series dilutions from 0 to 10×MIC of antibiotics was added into cultures of SA113 at OD_578_ = 0.1 in the BO medium. 0.5% xylose was added at OD_578_ = 0.5. OD_578_ was measured every hour. The final concentration was determined as the growth of bacteria was partially inhibited but still viable. The final concentrations used were: penicillin G 0.02 µg/ml (Serva, Heidelberg, Germany), moenomycin (flavomycin) 1 µg/ml (Sigma), bacitracin 2 µg/ml (Sigma), vancomycin 0.5 µg/ml (Sigma), tunicamycin 1 µg/ml (Serva, Heidelberg, Germany), gallidermin 0.1 µg/ml (Genmedics, Reutlingen, Germany), D-cycloserine 20 µg/ml (Sigma).

### Fluorescence microscopy

Cell wall and cross walls were visualized by fluorescence labeled vancomycin (BODIPY® FL vancomycin, Van-FL) staining [35] Cell samples taken at desired times were mixed with 1 µg/ml Van-FL (Invitrogen) and incubated for 5 min in the dark. 10 µl cell suspension was applied to the glass slide covered with 2% agarose. Fluorescent microscopy was performed with Leica DM5500 B Upright microscope. Images were captured with Leica DFC360 FX high-sensitivity monochrome digital camera. 504 ms exposure time was used for mCherry RF images. Fluorescence quantification was performed using Leica Application Suite Advanced Fluorescence software and ImageJ software.

## Supporting Information

Figure S1 **Quantification of visible cross walls and cross wall localized RF in the presence of penicillin or moenomycin. A.** Percentage of visible cross walls. The percentage was the ratio of visible cross wall numbers in a cell population versus the total cell numbers of the same cell population. Cross wall numbers were counted when Van-FL staining appeared as a line at the septum before daughter cells split (closed cross wall). More than 1000 cells from three independent experiments were counted. **B.** Percentage of cross wall localized RF. The percentage was the ratio of numbers of line-like cross wall localized RF versus line-like cross walls (visible by Van-FL staining) in the same cell population. The total cells numbers counted were above 1000 from three independent experiments for every bar. Statistical analysis was performed using Student's *t*-test. *P-*values of statistic analysis between treated and untreated cells (inter-group comparison) were marked above the bar of the corresponding treated group; *P*-values of intra-group comparison were marked on the horizontal line. *P<0.05, **P <0.01, ***P<0.005.
(TIF)

Figure S2 **Localization patterns of SA113 (pCXmCh-sec1&2) in the presence of penicillin or moenomycin.** Arrows in **b** and **e** indicted half-moon distribution of mCh-sec; arrows in **c** and **f** indicated dispersed mCh-sec over the entire cell; arrowheads, cross wall localized mCh-sec.
(TIF)

Figure S3 **Fluorescence intensity and Western blotting comparison between secreted GFP- and mCh-hybrids. A.** Fluorescence intensity of the culture supernatant from GFP/mCh-hybrids. The vertical axis indicated the ratio of the fluorescence intensity compared to the blank. **B.** Western blotting of the culture supernatant from GFP/mCh-hybrids. All of the GFP-hybrid plasmids were constructed in the same way as the mCh-hybrids and expressed in the protein A deficient mutant SA113 Δ*spa*. Blank, SA113 Δ*spa* without plasmid; GFP, SA113 Δ*spa* (pCX-*gfpmut3*); ppGFP, SA113 Δ*spa* (pCX-*pp_lip_gfpmut3*); ppGFP-sec1, SA113 Δ*spa* (pCX-*sp_lip_pp_lip_gfpmut3*); ppGFP-sec2, SA113 Δ*spa* (pCX-*sp_sasF_pp_lip_gfpmut3*); sfGFP, SA113 Δ*spa* (pCX-*sfgfp*); ppsfGFP, SA113 Δ*spa* (pCX-*pp_lip_sfgfp*); ppsfGFP-sec1, SA113 Δ*spa* (pCX-*sp_lip_pp_lip_sfgfp*); ppsfGFP-sec2, SA113 Δ*spa* (pCX-*sp_sasF_pp_lip_sfgfp*); ppmCh, SA113 Δ*spa* (pCXmCh-cyto); ppmCh-sec1, SA113 Δ*spa* (pCXmCh-sec1); ppmCh-sec2, SA113 Δ*spa* (pCXmCh-sec2). Arrows indicated the unprocessed (upper band) or the processed (lower band) form of the secreted GFP/mCh fusions.
(TIF)
