# Supplementary figures and images for: Cell Wall Antibiotics Provoke Accumulation of Anchored mCherry in the Cross Wall of Staphylococcus aureus
Source: PLoS One. 2012 Jan 10;7(1):e30076. doi: 10.1371/journal.pone.0030076 (PMC3254641; doi:10.1371/journal.pone.0030076)

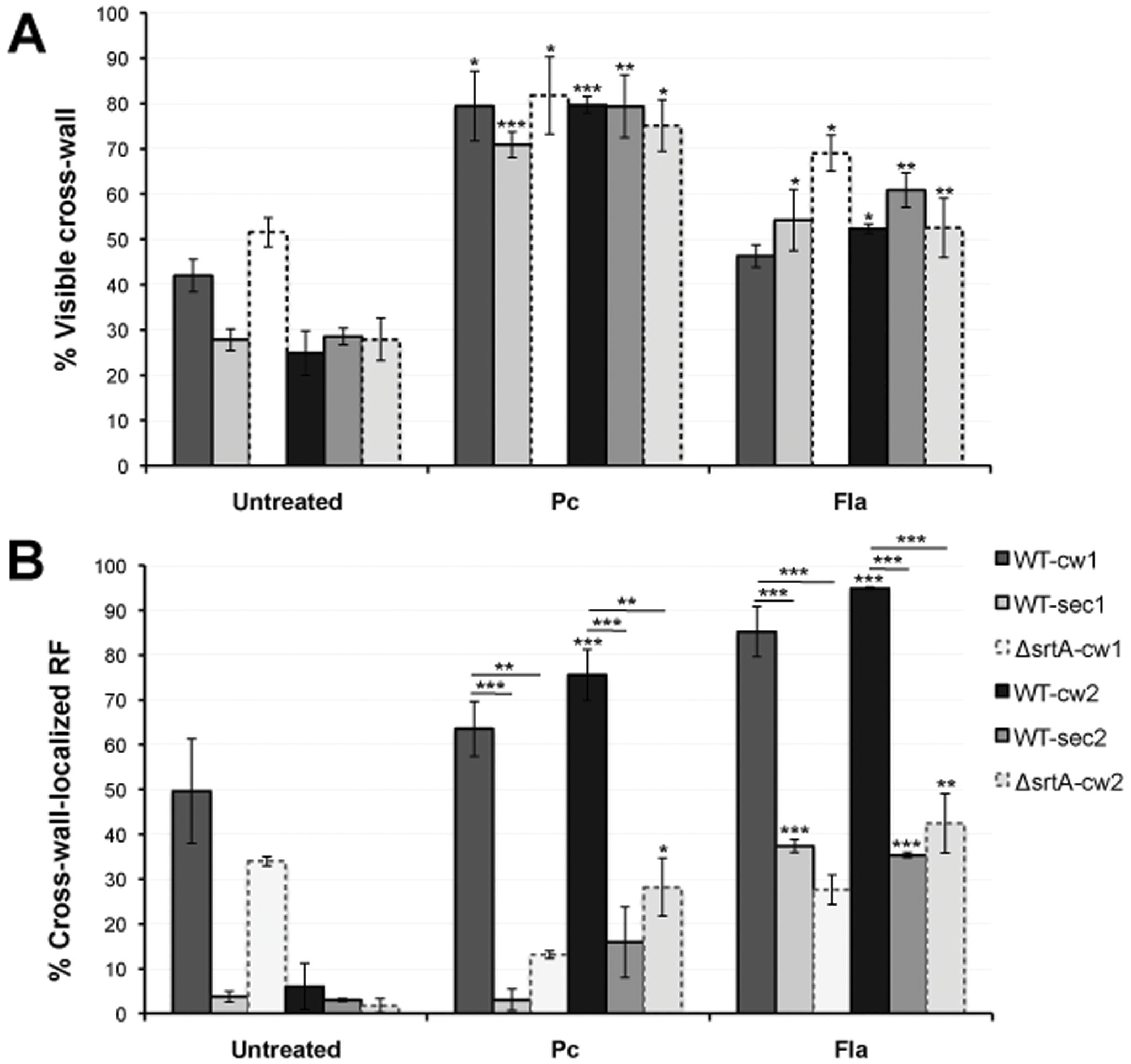

Supplement: Figure S1 — Quantification of visible cross walls and cross wall localized RF in the presence of penicillin or moenomycin. A. Percentage of visible cross walls. The percentage was the ratio of visible cross wall numbers in a cell population versus the total cell numbers of the same cell population. Cross wall numbers were counted when Van-FL staining appeared as a line at the septum before daughter cells split (closed cross wall). More than 1000 cells from three independent experiments were counted. B. Percentage of cross wall localized RF. The percentage was the ratio of numbers of line-like cross wall localized RF versus line-like cross walls (visible by Van-FL staining) in the same cell population. The total cells numbers counted were above 1000 from three independent experiments for every bar. Statistical analysis was performed using Student's t-test. P-values of statistic analysis between treated and untreated cells (inter-group comparison) were marked above the bar of the corresponding treated group; P-values of intra-group comparison were marked on the horizontal line. *P<0.05, **P <0.01, ***P<0.005. (TIF) [file pone.0030076.s001.tif]

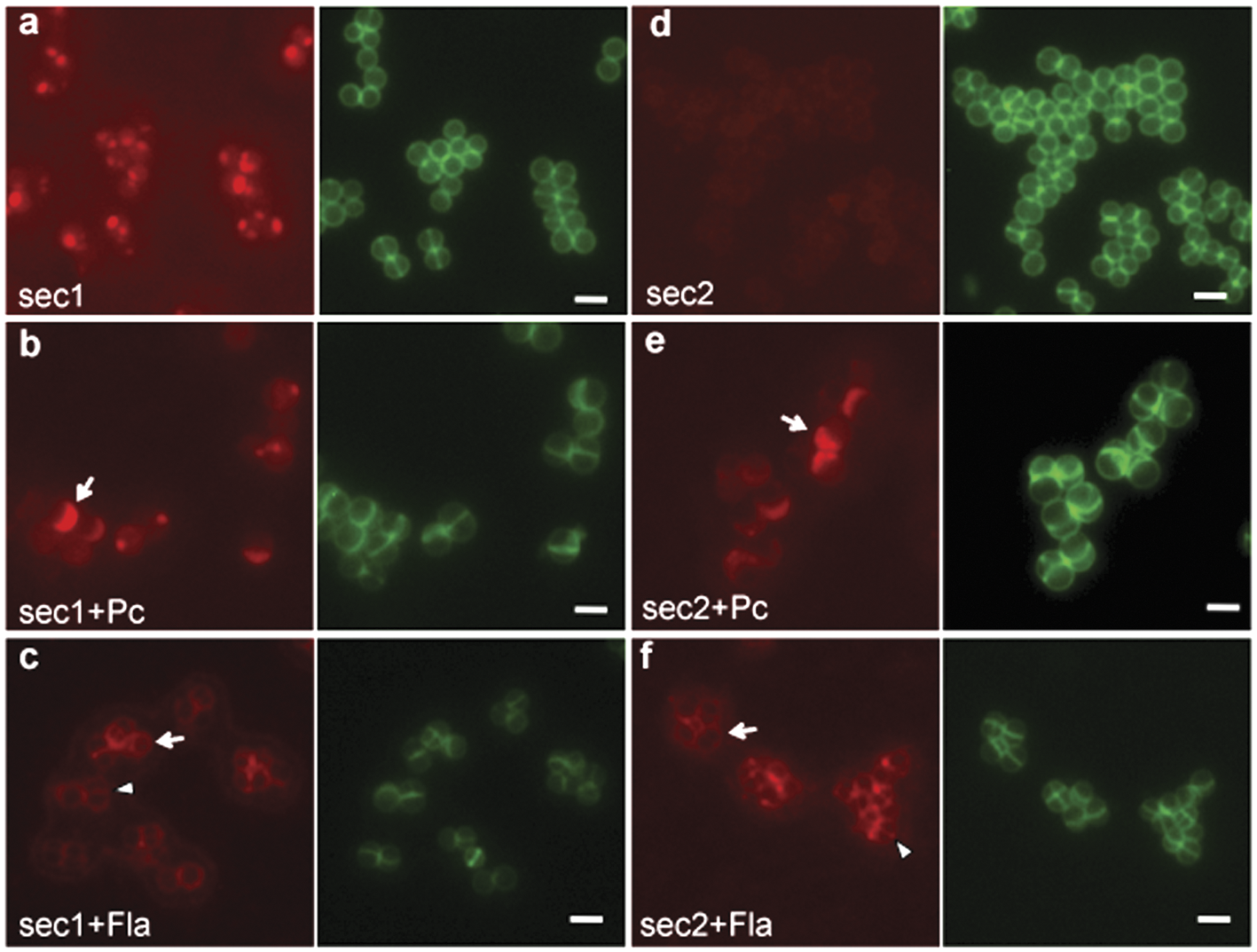

Supplement: Figure S2 — Localization patterns of SA113 (pCXmCh-sec1&2) in the presence of penicillin or moenomycin. Arrows in b and e indicted half-moon distribution of mCh-sec; arrows in c and f indicated dispersed mCh-sec over the entire cell; arrowheads, cross wall localized mCh-sec. (TIF) [file pone.0030076.s002.tif]

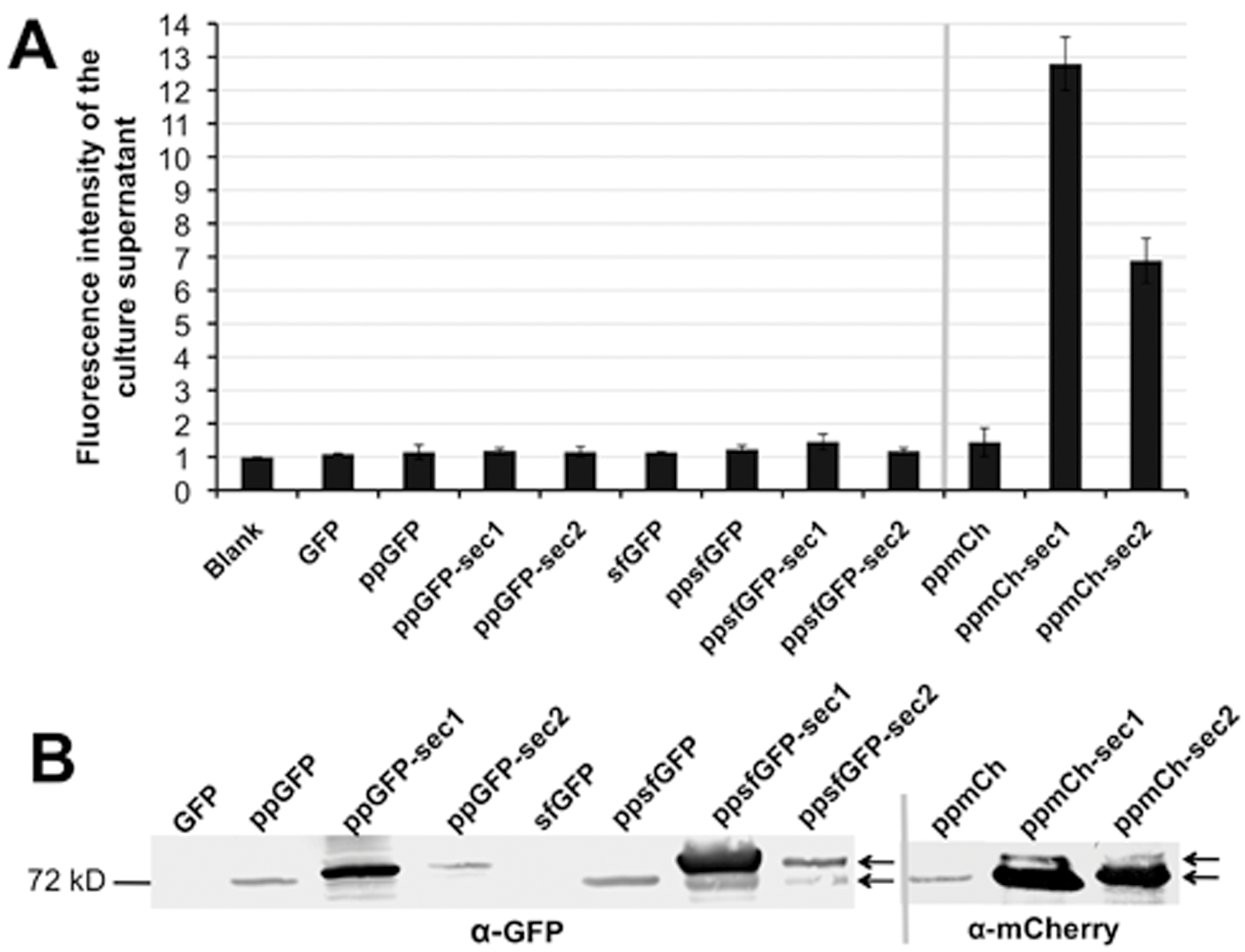

Supplement: Figure S3 — Fluorescence intensity and Western blotting comparison between secreted GFP- and mCh-hybrids. A. Fluorescence intensity of the culture supernatant from GFP/mCh-hybrids. The vertical axis indicated the ratio of the fluorescence intensity compared to the blank. B. Western blotting of the culture supernatant from GFP/mCh-hybrids. All of the GFP-hybrid plasmids were constructed in the same way as the mCh-hybrids and expressed in the protein A deficient mutant SA113 Δspa. Blank, SA113 Δspa without plasmid; GFP, SA113 Δspa (pCX-gfpmut3); ppGFP, SA113 Δspa (pCX-pplipgfpmut3); ppGFP-sec1, SA113 Δspa (pCX-splippplipgfpmut3); ppGFP-sec2, SA113 Δspa (pCX-spsasFpplipgfpmut3); sfGFP, SA113 Δspa (pCX-sfgfp); ppsfGFP, SA113 Δspa (pCX-pplipsfgfp); ppsfGFP-sec1, SA113 Δspa (pCX-splippplipsfgfp); ppsfGFP-sec2, SA113 Δspa (pCX-spsasFpplipsfgfp); ppmCh, SA113 Δspa (pCXmCh-cyto); ppmCh-sec1, SA113 Δspa (pCXmCh-sec1); ppmCh-sec2, SA113 Δspa (pCXmCh-sec2). Arrows indicated the unprocessed (upper band) or the processed (lower band) form of the secreted GFP/mCh fusions. (TIF) [file pone.0030076.s003.tif]
